# Acute neuro-athletic training effects on cognitive–motor and technical performance in youth basketball players

**DOI:** 10.3389/fnbeh.2026.1791909

**Published:** 2026-04-08

**Authors:** Oguzhan Tuncel, Resat Sadik, Oguzhan Yuksel, Aliye Buyukergun Kaplan, Ali Polat Cakici, Ceren Sevval Karatas, Gorkem Acar, Caglar Soylu

**Affiliations:** 1 Department of Coaching Education, Faculty of Sport Sciences, Igdır University, Iğdır, Türkiye; 2 Faculty of Sport Sciences, Duzce University, Düzce, Türkiye; 3 Department of Recreation, Faculty of Sport Sciences, Kutahya Dumlupinar University, Kütahya, Türkiye; 4 Department of Coaching Education, Faculty of Sports Sciences, Istanbul Topkapi University, Istanbul, Türkiye; 5 Department of Physical Education and Sports, Institute of Health Sciences, Balikesir University, Balıkesir, Türkiye; 6 Gulhane Institute of Health Sciences, University of Health Sciences, Ankara, Türkiye; 7 Faculty of Sport Sciences, Istanbul Gelisim University, Istanbul, Türkiye; 8 Gulhane Faculty of Physiotherapy and Rehabilitation, University of Health Sciences, Ankara, Türkiye

**Keywords:** acute performance, basketball, neuro-athletic training, visuomotor integration, youth athletes

## Abstract

**Background:**

Basketball performance emerges from the rapid integration of sensory information, motor execution, and technical skill. Neuro-athletic training (NAT) has gained attention as a sensory-driven intervention targeting visuomotor, vestibular, and proprioceptive systems to acutely enhance performance. However, evidence regarding the immediate and short-term effects of a single NAT session in youth basketball players remains limited.

**Objectives:**

To investigate the acute and short-term (30 min) effects of a single-session neuro-athletic training intervention on physical and basketball-specific technical performance in male youth basketball players.

**Methods:**

Forty-two male youth basketball players (14–17 years) completed a single-group repeated-measures study. Participants performed a single-session neuro-athletic training (NAT) protocol consisting of three stations integrating visual tracking, near–far focusing, reaction-based tasks, gaze stabilization, and basketball-specific skills such as passing, dribbling, and shooting. Assessments were conducted at baseline (Pre), immediately after NAT (Immediate), and 30 min post-intervention (Post-30). Outcomes included sit-and-reach flexibility, countermovement jump (CMJ) height, 20-m sprint time, dynamic balance, and AAHPERD passing and shooting tests.

**Results:**

Significant time effects were observed for all outcomes (all *p* < 0.001). Sit-and-reach performance increased from 7.69 ± 7.86 cm at Pre to 9.31 ± 7.67 cm immediately after NAT and 9.62 ± 7.79 cm at 30 min (η^2^_*p*_ = 0.508). CMJ height increased from 25.09 ± 5.25 to 27.66 ± 5.23 cm immediately and 28.60 ± 5.33 cm at 30 min (η^2^_*p*_ = 0.581), whereas 20-m sprint time decreased from 1.80 ± 0.30 to 1.62 ± 0.25 s immediately and remained lower at 1.74 ± 0.29 s at 30 min (η^2^_*p*_ = 0.425). Passing and shooting scores also improved markedly, increasing from 28.38 ± 3.04 to 34.62 ± 3.06 and 36.19 ± 2.99 (η^2^_*p*_ = 0.870), and from 16.00 ± 3.20 to 20.81 ± 3.42 and 22.81 ± 3.56 (η^2^_*p*_ = 0.793), respectively.

**Conclusion:**

A single-session neuro-athletic training intervention induced rapid and meaningful improvements in physical and basketball-specific technical performance, with several benefits retained after 30 min. These findings support NAT as an effective acute priming strategy for youth basketball performance.

## 1 Introduction

Basketball is a high-speed, open-skill team sport in which performance depends on the interaction of physical capacities, perceptual–cognitive processing, and rapid motor execution (Huynh et al., 2024; Jin et al., 2023; Piras, 2024; Lai et al., 2024). Players are required to process complex visual information, coordinate whole-body movements, and respond quickly to unpredictable environmental demands. Accordingly, basketball performance is influenced not only by strength, speed, and endurance, but also by visuomotor integration, postural control, and sensorimotor coordination (Buscemi et al., 2024; Lochhead et al., 2024).

Vision plays a central role in basketball-specific performance, supporting shooting accuracy, pass selection, defensive anticipation, and spatial decision-making (Jin et al., 2023; Vickers et al., 2017; Rösch et al., 2021; Christenson and Winkelstein, 1988). Recent evidence has shown that visual and visuomotor abilities are important contributors to performance in dynamic sport environments, with elite athletes demonstrating faster visual processing and more efficient perception–action coupling than non-athletes (Buscemi et al., 2024; Lephart et al., 2024; Lochhead et al., 2024; Hülsdünker et al., 2019). In addition, effective athletic movement depends on the integration of visual, vestibular, and proprioceptive inputs within the central nervous system (Cullen and Zobeiri, 2021).

Neuro-Athletic Training (NAT) has emerged as an approach aimed at improving brain–body interaction through exercises targeting visual, vestibular, and proprioceptive systems in combination with motor tasks (Appelbaum and Erickson, 2018; Soylu and Altundag, 2024). This approach is based on the premise that sensory input quality and neural processing may influence movement organization and sport-specific execution, consistent with principles of experience-dependent neural plasticity (Kleim and Jones, 2008). From an acute-performance perspective, brief preparatory interventions may act as neural priming strategies by temporarily enhancing central nervous system readiness before task execution (Moriarty et al., 2019). Experimental findings have also suggested that short interventions targeting sensorimotor processes may acutely influence balance, reaction time, and technical performance (Niederer et al., 2019).

At the same time, acute performance changes observed after a brief intervention may also reflect post-activation performance enhancement (PAPE), in which a prior high-intensity muscular activity transiently improves subsequent explosive or sport-related performance (Blazevich and Babault, 2019; Genç et al., 2025). Because both neural priming and PAPE may produce short-term performance improvements, this distinction should be considered when interpreting immediate post-intervention responses.

Recent sport-specific studies have reported beneficial effects of neuro-sensory and vision-based interventions on performance-related outcomes. Improvements in visuomotor skills and shooting performance have been reported following targeted vision training, and performance gains have also been observed after stroboscopic visual interventions in elite athletes (Cheatham et al., 2021; Lephart et al., 2024). However, evidence specific to basketball, particularly regarding the acute effects of a single NAT session, remains limited. Most existing studies have focused on longer-term interventions or on sports other than basketball (Flórez Gil et al., 2024; Soylu and Altundag, 2024; Cakici et al., 2026). In addition, little is known about whether any acute effects persist beyond the immediate post-intervention period.

This question may be particularly relevant in youth athletes, given the responsiveness of developing sensorimotor systems to training stimuli (Poltavski et al., 2021). However, empirical evidence examining both immediate and short-term retention effects of NAT in youth basketball players is currently lacking.

Therefore, the aim of the present study was to investigate the acute effects of a single-session neuro-athletic training protocol on physical, technical, and cognitive-motor performance in young male basketball players. Performance was assessed at baseline, immediately after the intervention, and 30 min post-intervention in order to evaluate both immediate and short-term responses. It was hypothesized that a single session of NAT would improve flexibility, balance, explosive power, sprint performance, and basketball-specific technical skills, and that these effects would be at least partially retained at 30 min.

## 2 Materials and methods

### 2.1 Participants

A total of 42 male youth basketball players (aged 14–17 years) completed all testing procedures and were included in the final analyses. Participants were recruited from organized youth basketball programs and were required to have a minimum training background consistent with competitive youth participation. Ethics approval for this study was granted by the Gülhane Scientific Research Ethics Committee of the University of Health Sciences, with approval number 2025-49, dated 7 January 2025. All participants provided written informed assent, and written informed consent was also obtained from parents/legal guardians prior to participation in accordance with the Declaration of Helsinki. Baseline anthropometric characteristics were recorded prior to testing (height, body mass, BMI) using standardized procedures.

Inclusion criteria were: (i) male basketball players aged 14–17 years; (ii) ≥3 years of licensed/organized basketball experience; (iii) regular basketball training ≥ 3 sessions/week; (iv) ability to fully understand and follow test instructions; and (v) completion of all measurement time points (Pre, Immediate, Post-30).

Exclusion criteria were: (i) any musculoskeletal injury (especially lower-extremity) or pain limiting sport participation within the previous 6 months; (ii) history of vestibular disorders (e.g., vertigo), uncontrolled balance disorders, or recent concussion; (iii) visual disorders not adequately corrected with lenses (e.g., severe uncorrected refractive error, strabismus affecting task execution); (iv) diagnosed neurological or psychiatric conditions; (v) known cardiovascular disease contraindicating high-intensity effort; (vi) concurrent participation in a structured rehabilitation program; and (vii) inability to complete the NAT session or any testing procedures safely and consistently.

### 2.2 Experimental procedure

**FIGURE 1:**
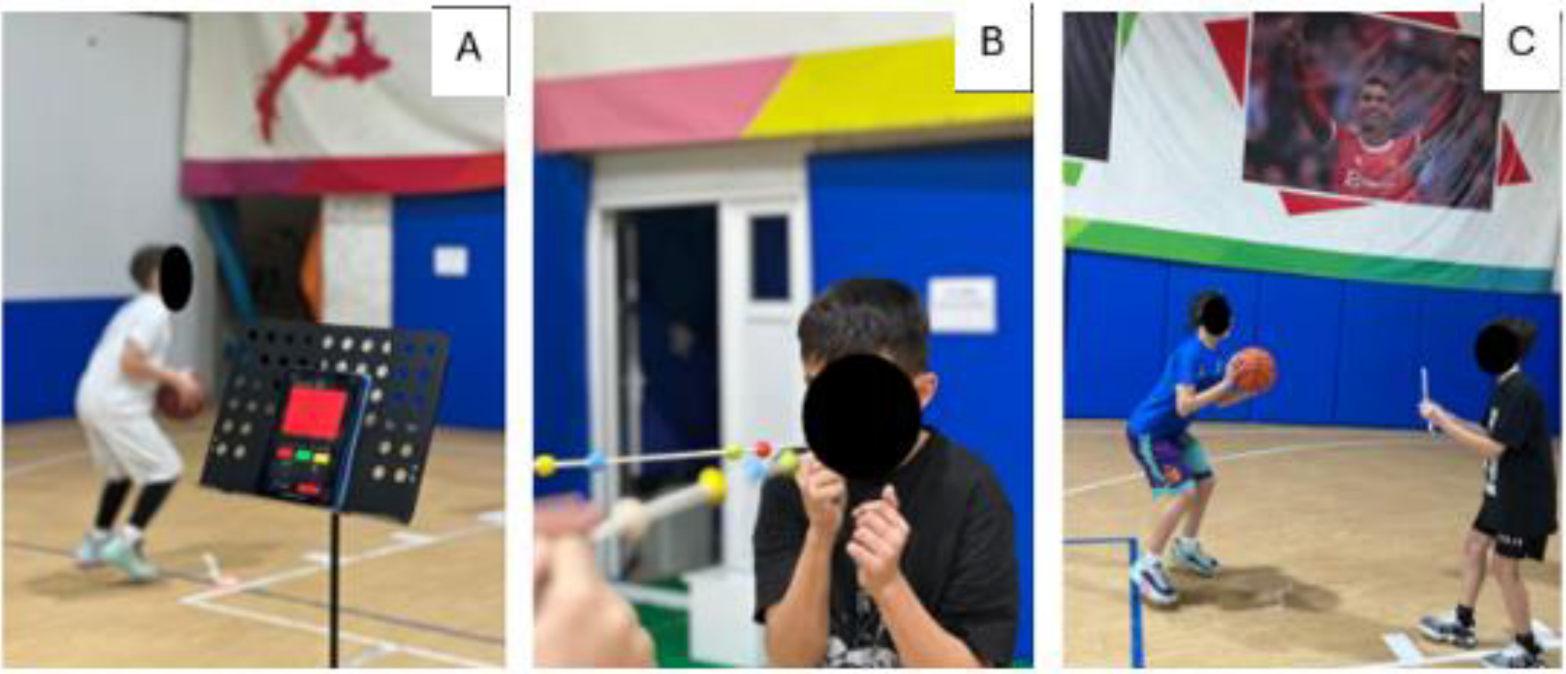
Representative examples of the neuro-athletic training (NAT) stations implemented during the intervention. The protocol integrated vision-based stimuli with basketball-specific motor tasks, including **(A)** visuomotor reaction training using an external visual cue system, **(B)** eye–hand coordination and defensive reaction drills with variable visual input, and **(C)** dynamic passing and shooting tasks performed under augmented perceptual constraints. All stations were designed to simultaneously challenge visual processing, sensorimotor integration, and technical execution in youth basketball players.

All data collection was performed at an indoor sports facility under stable environmental conditions. Participants attended a single laboratory/field-testing visit. After standardized briefing and familiarization with the procedures, baseline measurements (Pre) were obtained. All assessments were conducted in a fixed, non-randomized order within the same session, and the pre-intervention measurements served as the within-session control condition. Following completion of the baseline testing battery, participants completed the NAT protocol, after which the same outcome measures were repeated immediately after the session (Post-1) and again 30 min later (Post-30). A standardized recovery period of 3 min was provided between performance tests, and 1 min was allowed between repeated trials unless otherwise specified in the individual test procedures. During the Post-30 interval, athletes were instructed to avoid additional training stimuli and remained under supervision to standardize recovery conditions (Figure 1). No separate randomized or counterbalanced control session was performed; therefore, potential order and carry-over effects should be considered when interpreting the independent acute effect of NAT.

### 2.3 Neuro-athletic training (NAT) session

The acute NAT session was delivered as a structured, station-based protocol integrating vision-related and sensorimotor tasks with basketball-specific motor actions. The session lasted approximately 25 min and was implemented consistently for all participants under coach/researcher supervision. Standardized instructions, fixed work periods, and controlled transitions were used throughout the session. The protocol structure was developed according to applied neuroathletics practice resources and current sport-vision training concepts (Lienhard and Lienhard, 2018; Lienhard et al., 2019; Appelbaum and Erickson, 2018; Lochhead et al., 2024; Buscemi et al., 2024; Table 1).

**TABLE 1:** Neuro-athletic training protocol (single session): exercises, execution, and dose parameters.

Station	Exercise	Operational execution (how performed)	Dose (sets × reps/duration)	Standardization notes
1	Smart optometry + jump shot	Athlete completes a sport-vision tracking/drill on a screen/app (structured target tracking), then immediately receives a ball and performs a jump shot from pre-defined spots (right/left).	2 × 5 total jump shots	Same shooting distance and ball; same target task duration each set; consistent verbal cueing.
1	Cerebellum task (vision-stick letters + backward walk)	Dominant eye occluded; athlete reads letters on a vision stick while walking backward in a straight lane, maintaining safe pace and alignment.	2 × 5 passes (short bouts)	Lane marked; spotter present; same letter size/distance; stop if instability occurs.
1	Fitlight reaction + ball pickup + shot	Athlete responds to light stimuli based on a pre-taught rule (e.g., reversed color mapping). Correct response requires moving to the correct cone, collecting a ball, and completing a shot attempt.	3 × 10 reactions	Same cone distances; same rule set for all; false starts repeated after brief reset.
2	Near–far focus + pass (dominant eye occluded)	With dominant eye occluded, athlete performs near–far focus shifts while walking, then executes a targeted chest pass to a marked target.	2 × 5 sequences	Same target height/distance; standardized walking speed instruction; consistent occlusion method.
2	Pinhole glasses “ball steal” game	Small group drill wearing pinhole glasses; athletes attempt controlled ball-steal in a bounded area following safety rules (no contact beyond light touch).	3 × 1 min	Same area size; safety-first rule set; equal exposure time.
2	Head-laser tracking + shot	Athlete wears head-laser device; tracks a moving paper target with head-controlled laser; when target stops, athlete transitions to a standardized shot attempt.	3 × 10 (5 right/5 left)	Same target path; same shooting location; same left-right order.
3	Vertical pursuit + dribble	Athlete performs vertical pursuit (up–middle–down tracking) while simultaneously dribbling at controlled height; transitions maintained without losing control.	2 × 8 sequences	Same pursuit cadence; same ball; failed dribbles restarted.
3	Brock string + pass	Athlete performs Brock string fixation for ∼10 s, then immediately executes a targeted pass to a marked region.	3 × 5 (10 s Brock each)	Same Brock string setup; same pass distance; standardized instruction on fixation points.
3	Near–far chart + pass/shot	Athlete alternates focus between near and far letter charts then performs a rapid pass or shot following a standardized command.	2 × 8 sequences	Same chart distances/letter size; consistent cue timing and task order.

NAT, neuro-athletic training.

The NAT protocol consisted of three sequential stations designed to provide controlled neurocognitive and sensorimotor stimulation while preserving basketball-specific relevance. Station 1 primarily focused on visual tracking, visual discrimination, and reaction-based responses combined with shooting actions. Station 2 emphasized visual focusing, perceptual adjustment, and head–eye coordination integrated with passing, shooting, and competitive ball-control tasks. Station 3 focused on gaze control, fixation, and near–far visual adjustment combined with dribbling, passing, and rapid task execution.

Across all stations, the intervention was standardized by maintaining the same equipment, spatial arrangement, task order, cueing procedures, exposure times, and safety instructions for all participants. Detailed descriptions of the exercises performed at each station are presented in Table 1.

### 2.4 Outcome measures

#### 2.4.1 Flexibility: sit-and-reach test

Posterior chain flexibility was assessed using the sit-and-reach test conducted on a standardized sit-and-reach box. Participants sat with knees fully extended, feet placed flat against the measurement box, and hands overlapped while reaching forward in a slow, controlled manner to avoid ballistic movement. Three trials were performed with 15-s rest intervals between attempts, and the best score (cm) was retained for analysis (Mayorga-Vega et al., 2014). Recent systematic evaluations have further supported its utility as a global posterior-chain flexibility indicator when standardized procedures are applied (Miller et al., 2021).

#### 2.4.2 Explosive power: countermovement jump (CMJ) height

Lower-limb explosive power was assessed using the countermovement jump (CMJ) on an Optojump photoelectric cell system (Microgate, Bolzano, Italy). Participants performed a rapid downward countermovement followed by a maximal vertical jump, with their hands placed on the hips to minimize arm-swing contribution. Standardized verbal instructions were provided to ensure consistent technique across trials. Three maximal attempts were recorded with 1-min rest intervals between jumps, and the highest CMJ height (cm) was used for statistical analyses. Three maximal attempts were recorded with 1-min rest intervals, and the highest CMJ height (cm) was retained for analysis. CMJ height was derived from Optojump flight time using the standard flight-time equation $h=\frac{g\times t^{2}}{8}$, where *g* = 9.81 m/s^2^ and *t* represents flight time (s) (Ruf et al., 2024; Yamashita et al., 2020).

#### 2.4.3 Linear speed: 20-m sprint time

Acceleration and linear speed were assessed using a 20-m sprint test performed from a standing start. Sprint time was recorded using an electronic timing gate system (Optojump, Microgate, Bolzano, Italy), with standardized gate placement and a consistent start procedure applied for all participants. Participants completed three maximal sprints with 2-min passive recovery between trials, and the best 20-m time (s) was retained for analysis (Barrera et al., 2023).

#### 2.4.4 Dynamic postural control: Star Excursion Balance Test (SEBT)

Dynamic balance was assessed using the Star Excursion Balance Test (SEBT) performed on a non-instrumented testing grid marked on the laboratory floor, with reach distance measured using a seca 201 ergonomic measuring tape (seca GmbH & Co. KG, Hamburg, Germany). Participants performed the test barefoot with their hands placed on the hips to minimize upper-extremity compensation. The stance foot was positioned at the center of the grid, and reach distance was measured from the center point to the point of maximal toe contact. Limb length of the stance leg was measured in the supine position from the anterior superior iliac spine to the distal tip of the medial malleolus using the same measuring tape, and reach distances were normalized to limb length. A 15-s rest interval was allowed between trials and a 30-s transition period was provided between directions. The greatest normalized reach distance from the three successful trials was retained for statistical analysis for the anterior, posteromedial, and posterolateral directions separately; a composite score was not calculated. The SEBT is a reliable and valid measure for assessing dynamic postural control and lower-extremity neuromuscular function (Gribble et al., 2012).

#### 2.4.5 Basketball technical skill assessment: AAHPERD passing and shooting tests

Basketball-specific technical skills were assessed using the AAHPERD Passing Test and Speed Shot Shooting Test. In the Passing Test, participants performed two 30-s trials while moving between designated wall targets and executing chest passes; the scores from both trials were summed. Each chest pass that hit the target or target line was awarded two points, each pass contacting the area between targets was awarded 1 point, and 0 points were given for passes contacting other areas, for stepping on or over the restraining line, or for using a pass other than a chest pass. In the Speed Shot Shooting Test, participants performed two 60-s trials from standardized court spots; the scores from both trials were summed. Each successful basket was awarded two points, each unsuccessful shot that contacted the rim was awarded 1 point, and 0 points were given for air balls. A 2-min passive rest interval was provided between repeated trials for both technical tests. All tests were administered using standardized equipment, ball size, court dimensions, and identical verbal instructions (Tannoubi et al., 2023).

### 2.5 Statistical analysis

All statistical analyses were performed using IBM SPSS Statistics version 26.0 (IBM Corp., Armonk, NY, United States). Data are presented as mean ± standard deviation (SD). Normality and outliers were checked prior to analysis, and sphericity was assessed using Mauchly’s test; when violated, Greenhouse–Geisser correction was applied.

Acute changes across the three time points (Pre, Immediate, and 30 min) were analyzed using one-way repeated-measures ANOVA for each outcome. Mean differences, 95% confidence intervals, and Cohen’s *d*z values were reported for pairwise comparisons, while ANOVA effect sizes were expressed as partial eta squared (η^2^_*p*_).

In addition, standardized mean change (SMC) was calculated for the Pre-to-Immediate and Pre-to-30 min comparisons using the baseline standard deviation as the denominator: SMC = (Mean_post−Mean_pre)/SD_pre (Morris, 2000). The interpretation of effect magnitude followed Cohen’s conventional benchmarks, with values of 0.20, 0.50, and 0.80 representing small, medium, and large effects, respectively (Cohen, 1988). Statistical significance was set at *p* < 0.05. Statistical significance was set at *p* < 0.05.

## 3 Results

### 3.1 Participant characteristics

A total of 42 male youth basketball players completed all testing procedures and were included in the final analyses. Participants had a mean body height of 180.1 ± 3.9 cm, body mass of 69.8 ± 6.4 kg, and body mass index (BMI) of 21.3 ± 1.4 kg/m^2^. No missing data or protocol deviations were observed. All participants successfully completed the neuro-athletic intervention and all measurement sessions according to the study protocol.

### 3.2 Flexibility performance (sit-and-reach)

A significant main effect of Time was observed for sit-and-reach performance *F*(2, —) = 15.49, *p* < 0.001, η^2^_*p*_ = 0.508 (Table 2). *Post hoc* comparisons demonstrated a significant improvement immediately after the intervention compared with baseline (mean difference = +1.62 cm, *p* = 0.014), with this enhancement maintained and slightly augmented at 30 min post-intervention (mean difference = +1.94 cm vs. Pre, *p* = 0.004; Table 3).

**TABLE 2:** Performance outcomes (mean ± SD) and repeated-measures ANOVA results across time.

Outcome	Pre	Immediate	30 min	F (df1, df2)	*P*	Partial eta^2^
Sit-and-reach (cm)	7.69 ± 7.86	9.31 ± 7.67	9.62 ± 7.79	15.49 (2, 30)	<0.001	0.508
CMJ height (cm)	25.09 ± 5.25	27.66 ± 5.23	28.60 ± 5.33	20.78 (2, 30)	<0.001	0.581
20-m sprint time (s)	1.80 ± 0.30	1.62 ± 0.25	1.74 ± 0.29	11.09 (2, 30)	<0.001	0.425
Passing test score	28.38 ± 3.04	34.62 ± 3.06	36.19 ± 2.99	100.30 (2, 30)	<0.001	0.870
Shooting test score	16.00 ± 3.20	20.81 ± 3.42	22.81 ± 3.56	57.57 (2, 30)	<0.001	0.793

CMJ, countermovement jump; Pre, baseline; Immediate, 0–15 s post; 30 min, 30 min post; η^2^_p_, partial eta squared.

**TABLE 3:** Pairwise comparisons (paired *t*-tests) with Holm-adjusted *p*-values.

Outcome	Contrast (post−pre)	Mean diff	95% CI	t (df)	*P*	P (Holm)	Cohen dz	d_av
Sit-and-reach (cm)	Immediate**−**Pre	1.62	[0.51, 2.74]	3.10 (15)	0.007	0.014	0.78	0.20
Sit-and-reach (cm)	30 min**−**Pre	1.94	[0.90, 2.98]	3.98 (15)	0.001	0.004	0.99	0.25
Sit-and-reach (cm)	30 min**−**Immediate	0.31	[0.06, 0.57]	2.61 (15)	0.020	0.020	0.65	0.04
CMJ height (cm)	Immediate**−**Pre	2.56	[0.38, 4.73]	2.51 (15)	0.024	0.034	0.63	0.44
CMJ height (cm)	30 min**−**Pre	3.51	[1.65, 5.37]	4.03 (15)	0.001	0.003	1.01	0.61
CMJ height (cm)	30 min**−**Immediate	0.96	[0.20, 1.71]	2.69 (15)	0.017	0.034	0.67	0.15
20-m sprint time (s)	Immediate**−**Pre	**−**0.18	[**−**0.25, **−**0.10]	**−**5.00 (15)	<0.001	<.001	**−**1.25	**−**0.57
20-m sprint time (s)	30 min**−**Pre	**−**0.05	[**−**0.10, **−**0.00]	**−**2.27 (15)	0.038	0.038	**−**0.57	**−**0.17
20-m sprint time (s)	30 min**−**Immediate	0.13	[0.06, 0.20]	3.79 (15)	0.002	0.004	0.95	0.40
Passing test score	Immediate**−**Pre	6.25	[4.70, 7.80]	8.59 (15)	<0.001	<0.001	2.15	2.09
Passing test score	30 min**−**Pre	7.81	[6.35, 9.27]	11.41 (15)	<0.001	<0.001	2.85	2.60
Passing test score	30 min**−**Immediate	1.56	[0.98, 2.15]	5.72 (15)	<0.001	<0.001	1.43	0.53
Shooting test score	Immediate**−**Pre	4.81	[3.06, 6.57]	5.85 (15)	<0.001	<0.001	1.46	1.52
Shooting test score	30 min**−**Pre	6.81	[4.73, 8.89]	6.98 (15)	<0.001	<0.001	1.75	2.07
Shooting test score	30 min**−**Immediate	2.00	[1.22, 2.78]	5.48 (15)	<0.001	<0.001	1.37	0.61

CI, confidence interval; dz, standardized mean change (paired); d_av, effect size using averaged SD; Holm, Holm–Bonferroni adjustment.

### 3.3 Explosive power (countermovement jump height)

Countermovement jump (CMJ) height demonstrated a pronounced main effect of Time *F*(2, —) = 20.78, *p* < 0.001, η^2^_*p*_ = 0.581 (Table 2). CMJ height increased significantly from baseline to the immediate post-intervention assessment (mean difference = +2.56 cm, *p* = 0.034), with a further significant increase observed 30 min after the intervention (mean difference = +3.51 cm vs. Pre, *p* = 0.003; Table 3). Effect size analyses revealed a moderate standardized mean change at 30 min post-intervention (SMC = 0.68; Table 4).

**TABLE 4:** Standardized mean change (SMC) effect sizes for pre- to post-intervention performance outcomes.

Outcome	Post timepoint	Mean change (post−pre)	SMC (d)	Effect magnitude
Sit-and-reach (cm)	Immediate	1.62	0.21	Small
Sit-and-reach (cm)	30 min	1.94	0.25	Small
CMJ height (cm)	Immediate	2.56	0.49	Small
CMJ height (cm)	30 min	3.51	0.68	Moderate
20-m sprint time (s)	Immediate	**−**0.18	**−**0.61	Moderate
20-m sprint time (s)	30 min	**−**0.05	**−**0.17	Trivial
Passing test score	Immediate	6.25	2.06	Very large
Passing test score	30 min	7.81	2.58	Very large
Shooting test score	Immediate	4.81	1.50	Very large
Shooting test score	30 min	6.81	2.13	Very large

SMC, standardized mean change; SD_pre, standard deviation at baseline.

### 3.4 Linear sprint performance (20-m sprint time)

A significant Time effect was detected for 20-m sprint performance *F*(2, —) = 11.09, *p* < 0.001, η^2^_*p*_ = 0.425 (Table 2). Sprint time decreased substantially immediately following the intervention compared with baseline (mean difference = −0.18 s, *p* < 0.001; Table 3), reflecting an acute improvement in acceleration capacity. At 30 min post-intervention, sprint time remained significantly faster than baseline (mean difference = −0.05 s, *p* = 0.038), although performance partially regressed compared with the immediate post-intervention value (mean difference = +0.13 s, *p* = 0.004; Table 3). Standardized mean change values indicated a moderate effect immediately post-intervention, which diminished to a trivial magnitude at 30 min (Table 4).

### 3.5 Passing performance

Passing test performance exhibited a very large main effect of Time *F*(2, —) = 100.30, *p* < 0.001, η^2^_*p*_ = 0.870 (Table 2). Significant improvements were observed immediately after the intervention (mean difference = +6.25 points, *p* < 0.001) and were further amplified at 30 min post-intervention (mean difference = +7.81 points vs. Pre, *p* < 0.001; Table 3). Standardized effect size metrics confirmed very large acute and retained effects (SMC = 2.06–2.58; Table 4).

### 3.6 Shooting performance

Shooting performance also demonstrated a significant main effect of Time *F*(2, —) = 57.57, *p* < 0.001, η^2^_*p*_ = 0.793 (Table 2). *Post hoc* analyses revealed significant improvements immediately post-intervention (mean difference = +4.81 points, *p* < 0.001), with additional gains evident at 30 min post-intervention (mean difference = +6.81 points vs. Pre, *p* < 0.001; Table 3). Effect size analysis showed very large standardized mean changes at both post-intervention time points (SMC = 1.50–2.13; Table 4).

## 4 Discussion

The present study examined the acute changes observed following a single-session neuro-athletic training (NAT) protocol in flexibility, explosive power, linear sprint performance, and basketball-specific technical skills in male youth basketball players. Overall, the results showed statistically significant improvements across the assessed outcomes at the immediate and/or 30-min post-intervention assessments.

### 4.1 Flexibility

Sit-and-reach performance improved significantly immediately after NAT and remained elevated at 30 min. In athletic and youth settings, the sit-and-reach test is widely used and sensitive to short-term flexibility changes when standardized procedures are followed (Mayorga-Vega et al., 2014). The observed pattern in the present study—immediate improvement with maintained or slightly augmented effects at 30 min—aligns with evidence that even short-duration stretching or neuromuscularly oriented stimuli can produce time-dependent improvements in flexibility measures (Miller et al., 2021). Accordingly, the observed flexibility changes should be interpreted cautiously and considered as being associated with the overall intervention sequence rather than as definitive evidence of a specific NAT-driven mechanism. Because the present design does not allow isolation of the independent effect of NAT, these short-term responses may reflect non-specific acute changes, such as altered stretch tolerance or neuromuscular modulation, rather than structural tissue adaptation (Weppler and Magnusson, 2010; Behm et al., 2023). From an applied perspective, the retention of flexibility gains at 30 min is particularly relevant for pre-competition preparation in basketball, where warm-up-to-tip-off gaps frequently occur and flexibility changes can influence movement quality and technical execution.

### 4.2 Explosive power

Countermovement jump height improved immediately after the intervention sequence and showed a further increase at 30 min. Because CMJ is a sensitive indicator of acute neuromuscular status and stretch–shortening cycle readiness (Markovic et al., 2004; Cormack et al., 2008; Claudino et al., 2017), this pattern may at first glance appear compatible with an acute priming response. However, the current experimental design does not allow the increase in CMJ performance to be clearly separated from alternative acute influences, particularly PAPE-related responses elicited by the preceding sprint and jump efforts included in the assessment sequence. In applied sport contexts, CMJ is commonly used to monitor neuromuscular status and acute potentiation-like responses to priming stimuli (Claudino et al., 2017). Therefore, although NAT may have contributed to improved jump performance, the present data cannot determine whether the observed changes were caused primarily by neuro-sensory stimulation, by prior high-intensity muscular activity, or by the combined influence of both.

### 4.3 Sprint performance

Sprint time decreased substantially immediately after NAT, indicating improved acceleration capacity in the acute phase. However, this improvement should be interpreted with caution. The performance benefit partially regressed by 30 min, although sprint time remained significantly better than baseline. This time course may be compatible with a short-lived acute enhancement effect, but the present design cannot establish whether this reflects NAT-specific sensorimotor facilitation or a more general priming/PAPE response resulting from the testing and movement sequence. While the current study was not designed to identify physiological mechanisms, the observed pattern suggests that NAT-induced sprint benefits may be most pronounced within a short post-intervention window and may require careful scheduling to align with performance needs.

### 4.4 Technical performance

The largest observed changes were found in passing and shooting scores, both immediately after the intervention and again at 30 min. These findings suggest that the intervention sequence was associated with improved performance in controlled basketball-specific skill tests. However, given the lack of a separate counterbalanced control session, these changes should not be interpreted as unequivocal evidence of a direct NAT effect. The magnitude and retention of these technical effects are particularly important given that basketball performance is heavily dependent on rapid visuomotor processing and precision under time constraints. Contemporary sport-vision literature emphasizes that athletic performance is influenced more by dynamic visual and visuomotor abilities than by static acuity alone, and that targeted visual training can improve visuomotor skill execution (Buscemi et al., 2024; Lochhead et al., 2024). In this context, the NAT protocol’s integration of visual tasks with basketball-specific motor actions is consistent with modern approaches to enhancing perception–action coupling and visuomotor efficiency through structured training (Appelbaum and Erickson, 2018; Lochhead et al., 2024).

The broader sports-vision and neuro-sensory training literature supports the plausibility of rapid performance modulation after targeted visual or multisensory stimulation (Poltavski et al., 2021; Buscemi et al., 2024; Lochhead et al., 2024). At the same time, task repetition itself may enhance performance in standardized field tests, particularly when participants become more efficient in pacing, targeting, and movement organization across repeated attempts.

While the AAHPERD passing and shooting tests provide standardized, objective field measures of basketball skill execution (Hopkins et al., 1984), it is also essential to interpret these outcomes as controlled technical performance rather than direct in-game effectiveness. Accordingly, the present results are better viewed as evidence that performance in structured technical tasks improved across the experimental sequence, rather than as definitive proof that NAT independently enhanced match-relevant basketball skill. Given basketball’s reliance on rapid perception–action coupling, even short-term improvements in technical accuracy and execution speed can be valuable when translated into training environments and pre-competition preparation. However, direct ecological transfer to competitive game situations remains to be established.

Across outcomes, the general pattern of improvement is broadly consistent with the conceptual premise that interventions targeting visual and multisensory processing may influence subsequent motor output and technical execution. Neurophysiological models emphasize predictive sensory processing and the integration of multimodal inputs for effective movement planning and execution (Cullen and Zobeiri, 2021).

From an applied perspective, the findings indicate that the overall pre-performance sequence used in this study—which included NAT followed by repeated performance assessment—was associated with improved outcomes at immediate and 30-min time points. This may be relevant for coaches and practitioners when designing warm-up and priming routines. However, because the independent contribution of NAT cannot be clearly separated from other acute influences, practitioners should avoid assuming that the present data establish a direct cause-and-effect relationship between NAT and improved performance. Second, because different outcomes peaked at different times (sprint strongest immediately; CMJ and technical skills showing strong retention or further gains at 30 min), coaches may tailor NAT placement depending on the primary performance goal. Even so, such recommendations should be considered preliminary until confirmed by randomized, counterbalanced, or sham-controlled studies. Third, the station-based integration of visual tasks with basketball-specific actions is consistent with best-practice principles from sports-vision training emphasizing task specificity and representative learning design (Appelbaum and Erickson, 2018; Buscemi et al., 2024; Lochhead et al., 2024).

Several limitations should be considered when interpreting these findings, and these limitations substantially constrain causal interpretation. First, the study used a fixed-order repeated-measures design in which the baseline condition functioned as the within-session control, but no separate randomized, counterbalanced, or sham control session was included. Although the within-subject changes were large and consistent across multiple outcomes, the absence of a control group limits causal attribution and does not fully rule out alternative explanations such as learning effects, repeated testing familiarity, or motivational influences. This limitation is particularly relevant for technical skill tests, where performance can improve across repeated exposures even within a single session. Second, because sprinting and jumping tasks were embedded within the testing sequence, the contribution of acute activation or PAPE-like responses cannot be excluded, particularly for CMJ and sprint outcomes. Thus, the present results should not be interpreted as demonstrating the isolated effect of NAT on explosive or speed performance. Third, while standardized procedures and familiarization were implemented, practice effects can still occur—especially for complex tasks requiring coordination and precision. The additional improvements at 30 min in passing and shooting could partly reflect learning or re-optimization with repeated attempts. Future studies should include a control group performing the same repeated testing without NAT, or a placebo/sham intervention, to isolate NAT-specific effects. Fourth, although rest intervals were standardized, the possibility remains that recovery dynamics interacted with prior test exposure and influenced the post-intervention outcomes. Fifth, the study assessed outcomes only up to 30 min post-intervention. While this time window is highly relevant for warm-up and pre-competition contexts, it remains unclear how long performance benefits persist beyond 30 min, and whether the magnitude would remain meaningful during full match play. Sixth, the use of field-based tests should not be viewed as a limitation in itself, since outcomes such as jump height and sprint time cannot be assessed during match play under controlled conditions. Instead, a more important limitation is that the subjective load of the intervention was not evaluated, which would have helped determine how closely the protocol resembled training or match demands from the players’ perspective. Finally, the study included only male youth athletes within a specific age and competitive profile. Neural responsiveness and training transfer may differ by sex, maturation status, training history, or competitive level, which limits generalizability to other basketball populations.

In summary, the present findings indicate that meaningful short-term performance improvements were observed after the intervention sequence employed in this study. However, these improvements should be interpreted as associations occurring within the tested protocol rather than as definitive evidence of an independent NAT effect. The results support the practical relevance of this line of inquiry, but stronger experimental designs are required before firm causal or mechanistic conclusions can be drawn.

## 5 Conclusion

In conclusion, a single-session neuro-athletic training intervention produced significant acute improvements in flexibility, countermovement jump height, sprint performance, and basketball-specific passing and shooting skills in male youth basketball players. Performance changes were evident immediately after NAT and, for several outcomes—particularly explosive power and technical skills—were retained or further enhanced 30 min post-intervention. These findings support the practical utility of NAT as an acute priming strategy that can be integrated into warm-up routines or pre-competition preparation in youth basketball. Future controlled trials should also include match-simulated intermittent performance protocols to better evaluate ecological transfer under repeated high-intensity team-sport demands (Nicholas et al., 2000).

## Data Availability

The original contributions presented in this study are included in this article/supplementary material, further inquiries can be directed to the corresponding authors.
